# Synergistic Effect of Anionic-Tuning and Architecture Engineering in BiPO_4_@C Anode for Durable and Fast Potassium Storage

**DOI:** 10.3390/molecules30030729

**Published:** 2025-02-06

**Authors:** Heying Chu, Yong Li, Yuanjie Liu, Xueping Chai, Hongzhou Zhang, Jingchuan Zhang

**Affiliations:** College of Mechanical and Electronic Engineering, Tarim University, Alar 843300, China; chuheying@taru.edu.cn (H.C.); deyuzhijia@163.com (Y.L.); 120050023@taru.edu.cn (Y.L.);

**Keywords:** bismuth phosphate, nanostructures, architecture engineering, anionic-tuning, potassium-ion batteries

## Abstract

Bismuth-based materials that adhere to the alloy/dealloy reaction mechanism are regarded as highly promising anode materials for potassium-ion batteries due to their high volume-specific capacity and moderate reaction potentials. However, their commercial viability has been limited by the effects of structural collapse due to volume distortion and impeded electron conduction, resulting in rapid capacity decline. In this work, a carbon-coated nanosized BiPO_4_ rod (BiPO_4_@C) was designed and fabricated to overcome the aforementioned challenges through the architecture engineering and anionic-tuning strategy. In particular, the nanosized nanorods significantly reduce the volume expansion; the incorporation of the bulk and open-skeleton anion PO_4_^3−^ serves to mitigate the considerable volume distortion and generates the high ionic conductivity product (K_3_PO_4_) to ameliorate the poor ionic transport due to the structural deformation. The elaborated BiPO_4_ rods exhibit high specific capacity (310.3 mAh g^−1^, at 500 mA g^−1^), excellent cycling stability (over 700 cycles at 500 mA g^−1^) and superior rate performance (137.8 mAh g^−1^, at 1000 mA g^−1^). Systematic ex-situ XRD and TEM, as well as kinetic tests, have revealed the “conversion-multistep alloying” reaction process and the “battery-capacitance dual-mode” potassium storage mechanism. Moreover, the thick electrodes showed excellent specific capacity and rate performance, demonstrating their significant application potential in the next generation of SIBs.

## 1. Introduction

The contradiction between the limited lithium resources (0.0017 wt% in the earth’s crust) and the rapidly expanding energy storage and electric vehicle markets has considerably constrained the further development of lithium-ion batteries (LIBs) [1]. Therefore, the development of new energy storage devices with low-cost and high-specific energy is an effective means to accelerate the development of the energy storage market. The abundance of potassium resources (2.09% in the earth’s crust), the maturity of the processing technology, the low electrochemical potential (K^+^/K: −2.93 V vs. E^o^), and the well-known rocking chair charging/discharging mechanism have collectively positioned potassium-ion batteries (PIBs) as a promising next-generation “beyond Li-ion” battery [2,3]. However, the process of matching the large radius of K^+^ (1.38 Å) with conventional electrode materials leads to problems such as slow ion migration and high volume strain [4,5]. Consequently, there is an urgent requirement to develop anode materials with a robust structure and a high ability to tolerate volume strain, as well as to obtain electrode materials with ultra-long cycle stability and rapid potassiation/depotassiation capabilities.

In terms of anode materials, the potassium storage capacity and cycling stability of graphite are much lower than that of commercial LIBs due to the drastic volume distortion that occurs during K^+^ insertion/extraction [6]. Metal-based materials (e.g., Sn [7,8], Sb [9,10], Bi [11,12], and binary/ternary alloys [13,14]) capable of alloying with K^+^ have attracted considerable attention due to their high conductivity, considerable theoretical specific capacity and safe redox potential. Among these materials, layered bismuth shows promising prospects for K^+^-storage due to its advantageous characteristics, including a high volumetric theoretical specific capacity (385 mAh g^−1^), non-toxicity, high electrical conductivity, large lattice spacing, and moderate discharge voltage [15]. However, the gradual alloying process with K^+^ inevitably causes significant volume strain (390%), which further leads to microstructural collapse and rapid deterioration of cycling performance [16]. Many strategies have been attempted to overcome the above problems, such as compositing with carbon media to mitigate the volume strain exerted on the active particles and reduce particle fragmentation [17,18], Construction of special micro-nanostructures to accommodate volume expansion and contraction through the incorporation of buffer spaces to avoid rupture of the outer stable solid electrolyte interphase (SEI) films [19,20]; Anion auxiliary strategies (Bi_2_O_3_ [21], Bi_2_S_3_ [22], Bi_2_Se_3_ [23], Bi_2_O_2_Se [24], Bi_2_Se_3−*x*_Se*_x_* [25]) to prevent agglomeration of metal elements and relieve structural stresses anchored by bonding with anions. Undeniably, the aforementioned subtle enhancement strategies have largely mitigated the damage to the electrode structure caused by volume distortion and significantly improved the cycling stability of Bi-based electrodes. Nevertheless, the scalability of this intricate process remains a significant challenge, and the difficulty of disrupting the electron conduction path during volume expansion and contraction has yet to be overcome. Furthermore, the poor conductivity of the discharge products of Bi-based chalcogenides and the strong evolution and shuttle effects (K_2_S and K_2_Se) in the anion tuning process are often overlooked, which are again the culprits for the capacity degradation of Bi-based electrodes.

Herein, a carbon-coated nanosized BiPO_4_ rod (BiPO_4_@C) was synthesized by a simple hydrothermal method to mitigate the drastic alloying reaction and improve ionic conductivity. Specifically, the small-sized nanorods reduce the transport distance of K^+^ and electrons within the solid phase, eliminate the size dependence, and mitigate the volume distortion. The introduction of the large-sized PO_4_^3−^ with a highly thermally stable and open P-O octahedral framework structure serves to buffer the significant volume distortion of the Bi anode during the alloying/dealloying process. Crucially, the discharge product (K_3_PO_4_) is generated in situ with high ionic conductivity, which boosts the electronic conductivity in damaged structures. Furthermore, the carbon coating improves the conductivity of the phosphate electrode and acts as an armor against structural degradation. As a consequence of these properties, the BiPO_4_@C electrode exhibits high specific capacity, excellent cycling stability and rate performance. Post-mortem analysis demonstrates the conversion-multistep alloying reaction mechanism. The kinetic tests verify the “battery-capacitor” dual-mode potassium storage behavior. Therefore, the synergistic strategy of anion tuning and nanosize effect in this work provides a promising avenue for the development of rapid and stable anodes for PIBs.

## 2. Results and Discussion

### 2.1. Synthesis and Structural Characterization

**Figure 1:**
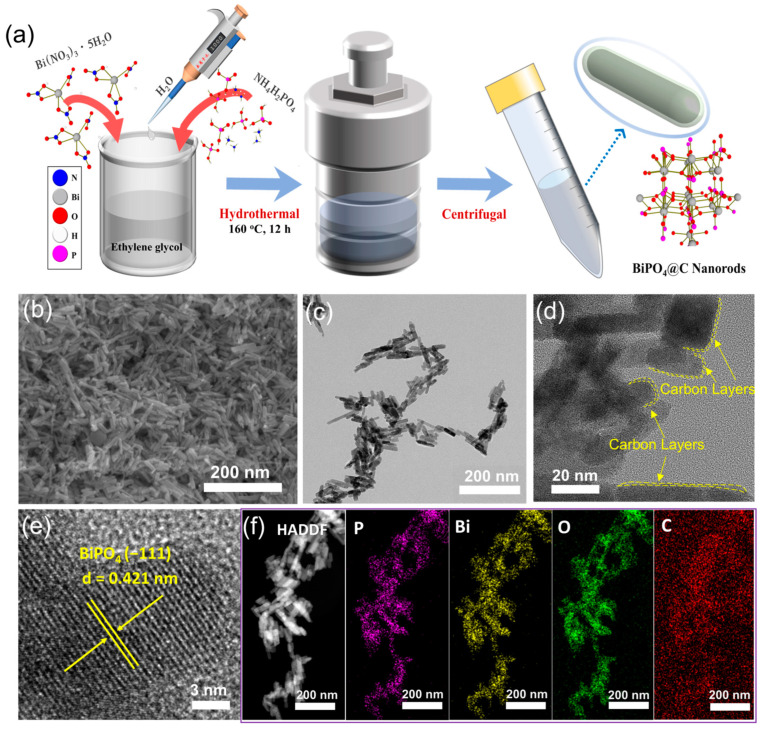
(**a**) Schematic of the fabrication process of BiPO_4_@C composite. (**b**) SEM, (**c**,**d**) TEM, (**e**) HRTEM images, and corresponding (**f**) HADDF and EDS mapping images of the obtained BiPO_4_@C.

Figure 1a illustrates a facile synthetic approach for BiPO_4_@C nanorod via a one-step hydrothermal process, in which Bi(NO_3_)_3_ and NH_4_H_2_PO_4_ were employed as the sources of Bi and P, respectively, while ethylene glycol (EG) was served as both the reaction medium and the carbon source. According to the literature, Bi^3+^ ions in solutions tend to be hydrolyzed to produce various hydroxides, depending on the composition of the solution [26]. Meanwhile, under mild heating, NH_4_H_2_PO_4_ molecules can be decomposed to form H_3_PO_4_. [27,28] Subsequent high temperature and pressure processes lead to the formation of BiPO_4_ nanorods. Based on the above analyses, we believe that the following reactions occur in the water/ethylene glycol mixture:

Bi(NO_3_)_3_ + 3H_2_O → Bi(OH)_3_ + 3HNO_3_ (1)

NH_4_H_2_PO_4_ → H_3_PO_4_ + NH_3_↑ (2)

Bi(OH)_3_ + H_3_PO_4_ → BiPO_4_ + 3H_2_O (3)

The microscopic morphology of BiPO_4_@C was investigated by scanning electron microscopy (SEM) and transmission electron microscopy (TEM). First, the effects of raw material concentration, reaction time and reaction temperature on the morphology of BiPO_4_@C composites were systematically investigated. As shown in Figures S1–S3, the diameter and length of BiPO_4_@C nanorods increase with increasing reaction concentration and reaction temperature, while the reaction time significantly restricts the morphology of the material. Taking all factors into consideration, we set the optimum raw material concentration, reaction time and temperature to 0.5 mmol, 160 °C, and 12 h, respectively. As shown in Figure 1b,c, BiPO_4_@C exhibits a nanosized rod-like structure with a length of approximately 50–80 nm and a diameter of 15–20 nm. Interestingly, as shown in Figure 1d, each BiPO_4_ nanorod exhibits a monocrystal structure with well-defined lattice fringes, and the surface of the nanorods is encapsulated with an ultra-thin carbon layer. Furthermore, the high-resolution TEM (HRTEM) image in Figure 1e clearly shows a set of periodic lattice fringes with a spacing of 0.421 nm, corresponding to the (−111) plane of monoclinic BiPO_4_ (PDF# 04-010-5606). Meanwhile, the energy dispersive spectroscopy (EDS) mapping results indicate that the elements Bi, P and O are distributed uniformly in the BiPO_4_@C nanorod composites (as shown in Figure 1f).

**Figure 2:**
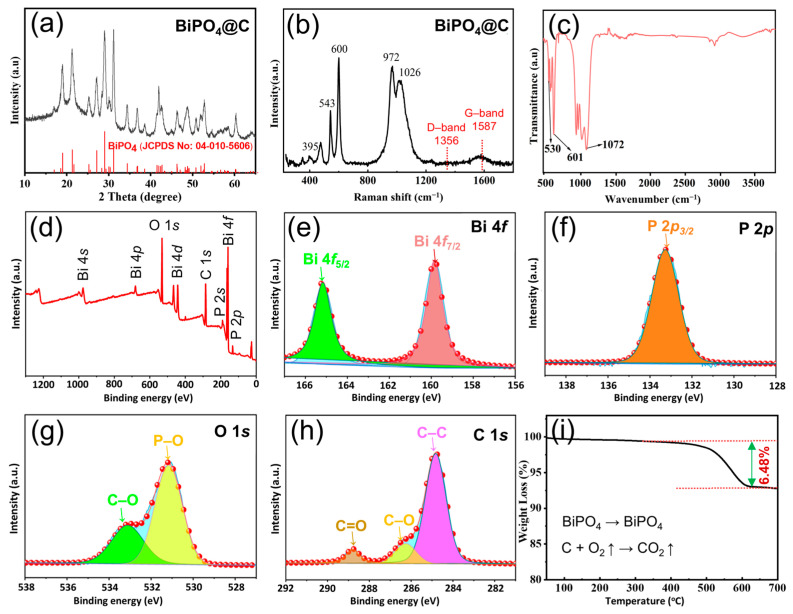
(**a**) XRD pattern, (**b**) Raman spectra, (**c**) FTIR spectra, (**d**) XPS survey spectra, high-resolution XPS spectra of (**e**) Bi 4f, (**f**) P 2p, (**g**) O 1s, (**h**) C 1s, and (**i**) TGA curve of BiPO_4_@C composite.

X-ray diffraction (XRD), Raman spectra, Fourier transform infrared spectroscopy (FTIR), and X-ray photoelectron spectroscopy (XPS) were used to determine the physical phase and structural information of BiPO_4_@C. As shown in Figure 2a and Figure S4 and Table S1, the XRD results indicated that the diffraction peaks of BiPO_4_@C were consistent with the pure phase of monoclinic BiPO_4_ (PDF# 04-010-5606). Subsequently, Raman spectra (Figure 2b) monitored a set of characteristic absorption peaks in the range of 300–1700 cm^−1^, wherein the peaks located at 395, 543, and 600 cm^−1^ can be ascribable to PO_4_^3−^, while the peaks appearing at 972 and 1026 cm^−1^ can ascribe to the symmetric (υ1) and asymmetric (υ2) scaling modes of PO_4_^3−^, respectively [29]. Two other characteristic peaks that appear at 1356 and 1587 cm^−1^ can be attributed to disordered graphitic carbon (D-bond) and sp^2^ hybridized graphic carbon (G-bond) of the C component, respectively. Obviously, a lower intensity ratio (I_D_/I_G_) value (0.37) for BiPO_4_@C indicates a regular graphitic structure and high electrical conductivity, which can significantly improve the poor electrical conductivity inherent in phosphate materials. Moreover, the FTIR spectra revealed the presence of chemical bonding information within the wavenumber range of 500 to 3700 cm^−1^ for BiPO_4_@C composites (as shown in Figure 2c). The results indicated that the absorption peaks at 530 and 601 cm^−1^ were indicative of the vibrational absorption of δ(PO_4_^3−^), while the absorption peak at 1072 cm^−1^ was attributed to the υ3 asymmetric stretching vibration of the P-O bond [30,31]. The three absorption peaks situated between 2750 and 2950^−1^ are ascribed to the symmetric and antisymmetric stretching vibrations of the C−H bond in the −CH_3_, while the vibrational absorption peaks of the benzenoid ring skeleton appear between 1459 and 1650 cm^−1^. Furthermore, the XPS survey spectrum clearly shows signals corresponding to the Bi, P, O, and C elements in BiPO_4_@C composites, as illustrated in Figure 2d. Specifically, the two peaks observed at 165.1 eV and 159.8 eV in the high-resolution Bi 4f can correspond to Bi 4f _5/2_ and Bi 4f _7/2_, respectively (Figure 2e) [12,29]. Additionally, the P 2p spectrum (Figure 2f) demonstrated the emergence of a prominent peak associated with the P 2p _3/2_ (at 133.7 eV) [29,32]. The high-resolution O 1s spectrum is split into two peaks at 531.2 and 533.1 eV, which are attributed to P-O and C-O bonds, respectively (Figure 2g) [29,32]. Additionally, we detected the characteristic peaks of the C=O (288.7 eV), C-O (286.3 eV), and C-C (284.8 eV) bonds in the C 1s spectrum, respectively, as shown in Figure 2h [33,34]. It is noteworthy that the presence of C-O bonding indicates that the ultrafine BiPO_4_ particles can be chemically bonded to form a linkage with the carbon layer, which will undoubtedly facilitate electron transfer and enhance the rate performance. Subsequently, the content of the carbon-protective layer in BiPO_4_@C was then determined by thermogravimetric analysis (TGA) test. As illustrated in Figure 2i, the mass remained constant up to 450 °C, after which a rapid mass loss (reduction of ~6.48 wt%) occurred in the range from 450 °C to 600 °C. In light of the TGA result and the deep oxidation products (BiPO_4_, as shown in Figure S5), the calculated contents of the product BiPO_4_ and carbon matrix in BiPO_4_@C composite are 93.58 and 6.42%, respectively. A small amount of carbon layer with high graphitization can improve the conductivity of BiPO_4_, alleviate the volume effect in the electrochemical process, and further improve the potassium-ion storage performance of BiPO_4_@C electrode without excessively reducing the specific capacity of the active component.

### 2.2. Electrochemical Properties

**Figure 3:**
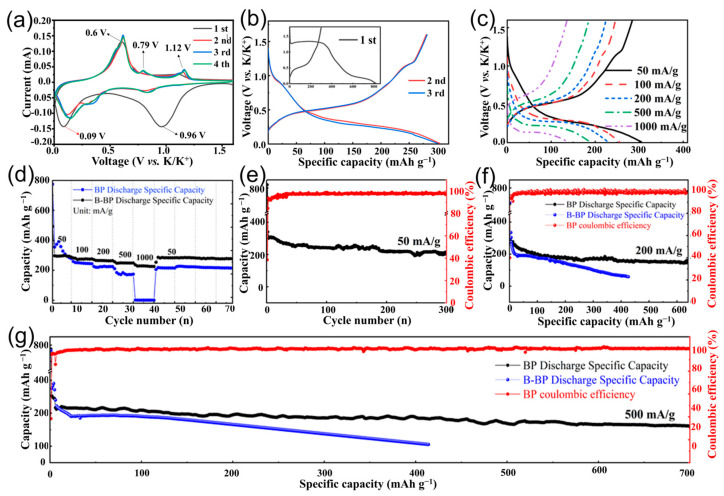
(**a**) CV curves at 0.1 mV s^−1^ and (**b**) galvanostatic charge/discharge curves at 0.2 A g^−1^ of the BiPO_4_@C electrode. (**c**) Cycling performances at 50 mA/g. (**d**) Rate performances and (**e**) corresponding discharge/charge profiles at various current densities of BiPO_4_@C electrode. Long cycling performances at (**f**) 200 mA/g and (**g**) 500 mA/g.

The potassium ion storage performance of BiPO_4_@C ultrafine nanorods and reference sample (commercial micro-sized BiPO_4_, morphology and crystalline phase are shown in Figure S6) was investigated in button-type half-cells and coupled with potassium metal as the counter electrode. Figure 3a depicts the cyclic voltammetry (CV) curves of the BiPO_4_@C electrode within the voltage range of 0.01 to 1.6 V at a scan rate of 0.1 mV s^−1^. A reduction peak at 0.96 V was observed in the initial cathodic scan, corresponding to the conversion reaction to produce Bi element and K_3_PO_4_ (BiPO_4_ + 3 K^+^ + 3 e^−^ → K_3_PO_4_ + Bi), as well as the formation of SEI layer [31]. Furthermore, a subsequent peak located at 0.09 V is associated with the alloying reaction to produce K_3_Bi (3 Bi + K^+^ + e^–^ → K_3_Bi). Subsequently, three anodic peaks located at 0.60, 0.79, and 1.12 V were detected due to the gradual dealloying reaction (K_3_Bi → K_x_Bi → Bi) [29]. During successive scans, the reduction peak at 0.96 V disappeared, suggesting an irreversible transformation process. Undoubtedly, the high ionic conductivity of K_3_PO_4_ is always maintained around the Bi particles, thereby accelerating the K^+^ conduction kinetics by repairing the ion transport channels. In subsequent cycles, the electroreception process of the Bi-metal gradually evolved into a unique, multistep alloy/dealloy process, exhibiting three pairs of oxidation/reduction peaks at 1.12/0.84, 0.79/0.35 and 0.6/0.14 V, respectively. [29,31]. Comfortingly, the alloying/dealloying reaction is highly reversible in terms of the positions and shapes of the CV curves, indicating the strong structural stability of BiPO_4_@C. Furthermore, the voltage platforms in the galvanostatic charge-discharge (GCD) curve (Figure 3b) are well consistent with the redox peaks observed in the CV curves, confirming the proposed mechanism of initial “conversion-alloying/dealloying” and the subsequent “alloying–dealloying” reactions.

Additionally, the BiPO_4_@C electrode displays a low polarization potential (Figure 3c) and excellent rate performance (Figure 3d). Accordingly, the specific charging capacities at 50, 100, 200, 500 and 1000 mA g^−1^ current density were 307.2, 254.9, 229.5, 187.9 and 137.8 mAh g^−1^, respectively, which are significantly higher than those of commercial BiPO_4_ electrodes and other reported Bi-based compound anode materials (Figure S7) [35,36,37,38,39]. The superior rate performance can be attributed to the short and efficient ion transport path provided by the nanostructuring effect and the accelerated ion transport kinetics provided by the fast ion conductor intermediate (K_3_PO_4_). It is encouraging to note that following multiple current shocks, the BiPO_4_@C electrode still exhibited a reversible capacity of 278.2 mAh g^−1^ when the current density was returned to 50 mA g^−1^, substantiating the fast electron/ion transport capacity and robust structure of the BiPO_4_@C electrode. Subsequent cycling performance tests highlighted the high specific charge capacity and exceptional electrochemical stability of the BiPO_4_@C electrode. As illustrated in Figure 3e, the BiPO_4_@C electrode exhibits an initial charge capacity of 310.3 mAh g^−1^ and reaches a specific capacity of 216 mAh g^−1^ after 300 cycles at a current density of 50 mA g^−1^ with a slower average decay rate of 0.101% per cycle. Not surprisingly, the reversible capacities of the BiPO_4_@C electrode after 600 cycles at 200 and 500 mA g^−1^ were an astonishing 161 mAh g^−1^ and 142 mAh g^−1^, respectively (Figure 3f,g). Nevertheless, the specific capacitance of the micro-sized BiPO_4_ electrode rapidly decreases due to the fact that the unprotected large-area structure cannot accommodate the large volume distortion that occurs during repeated alloying and dealloying. In addition, even at a current density of 1000 mA/g, the BiPO_4_@C electrode demonstrated stable operation over 150 cycles, maintaining a specific capacity of 171 mAh g^−1^ (Figure S8). These superior reversibility rate properties far exceed those of some previously reported phosphate anode electrodes [32,40,41].

To elucidate the robust cycle stability of the BiPO_4_@C electrode, we added the structural tests of the electrode surface and cross-section with multiple sets and performed a statistical analysis of the volume expansion. As expected, the BiPO_4_@C electrode surface retained an intact structure even after 100 cycles (Figure S9a,b), whereas commercial micro-sized BiPO_4_ electrodes exhibited obvious structural fragmentation (Figure S10a,b). Figure S9c,d shows the cross-sectional changes of the BiPO_4_@C electrodes before and after cycling. Specifically, the thickness of the BiPO_4_@C electrode is 20.31 µm before potassium insertion. After repeated potassiation/depotassiation, the thickness increases to 29.74 µm, and the corresponding volume expansion rate is 46.4%. In contrast, the commercial micro-sized BiPO_4_ electrode showed a volume expansion of 95.5%, and the electrode showed obvious signs of loosening and cracking (Figure S10c,d). It is clear that the BiPO_4_@C electrode, which combines the nanoscale effect and carbon-constrained structure, has significant resistance to volume expansion, which is the key to achieving stable potassium-ion storage. In addition, Electrochemical Impedance Spectroscopy (EIS) tests were carried out under different cycle conditions (Figure S11). Clearly, at high frequencies, the BiPO_4_@C electrode has a smaller semicircle, which means a lower impedance. Especially after the formation of the SEI film during the first cycle, the impedance decreases significantly and gradually stabilizes even after 50 to 100 cycles (Figure S11a). In contrast, the BiPO_4_ electrode consistently showed a large semicircle (Figure S11b), which can be attributed to the large interfacial impedance due to continuous volume expansion and electrode cracking. Overall, further testing confirms that the BiPO_4_@C electrode combines both nanoscale dimensions and carbon layer properties, is highly resistant to the volume strain inherent in alloy–dealloy reactions, achieves a robust structure and significantly improves cycle stability.

### 2.3. Kinetic Analysis

**Figure 4:**
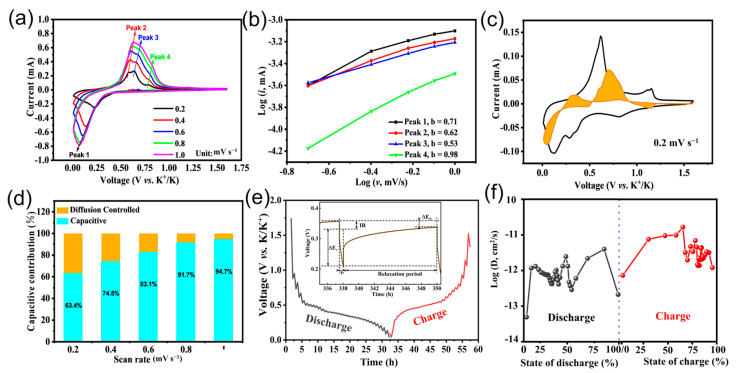
(**a**) CV curves at various scan rates, (**b**) the relationship between log (i) and log (v), and (**c**,**d**) contribution ratios of the capacitive-controlled capacity of the BiPO_4_@C electrode. (**e**) GITT curves and (**f**) the calculated potassium-ion diffusion coefficients of the BiPO_4_@C.

To ascertain the origin of the excellent rate performance of the BiPO_4_@C electrode, further tests were carried out on the kinetic and pseudocapacitive behavior. In detail, as depicted in Figure 4a, the five CV curves obtained at different sweep rates (0.1, 0.2, 0.6, 0.8, and 1.0 mV/s) show similar profiles and regular change trends of the redox peaks. The capacitive effect is calculated from the relationship between the measured peak current (*i*) and the sweep rate (*v*) (*i* = a*v*^b^), where *b* is the slope of the plot of log *i* versus log *v*. The atypical diffusion-controlled process corresponds to *b* = 0.5, while *b* = 1.0 indicates surface induced capacitive behavior. Furthermore, Figure 4b shows that the *b* values of the several peaks are distributed between 0.61 and 0.75, suggesting that the BiPO_4_@C electrode follows a “battery-capacitance dual-mode” potassium storage mechanism dominated by surface capacitive behavior. Moreover, the proportion of capacitance-controlled and diffusion-controlled processes contributing to the total specific capacity can also be quantified according to the equation of *i* = k_1_*v*+ k_2_*v*^1/2^, where k_1_*v* and k_2_*v*^1/2^ are to be assigned as the relative contributions of the capacitive and intercalation processes, respectively. As expected, the capacitive percentage was observed to be 31, 31, 43, 49 and 60% at scanning rates of 0.1, 0.2, 0.6, 0.8, and 1.0 mV s^−1^, respectively (Figure 4c,d). The high capacitive behavior is primarily attributable to the abundant surface reaction sites afforded by the ultrafine structure of BiPO_4_@C and the adsorption/desorption capacity of K^+^ by the ultrathin carbon layer, undoubtedly resulting in fast K-ion storage kinetics and ultralong cycling life. Meanwhile, the galvanostatic intermittent titration technique (GITT) measurements were carried out to identify the underlying causes of the superior K-ion storage kinetics obtained in the BiPO_4_@Celectrode. As depicted in Figure 4e, the GITT curve of the BiPO_4_@C electrode exhibits a high specific capacity, low electrochemical polarization and standard redox potential. Moreover, from the pulse current time (s), potential difference (Δ*E*_S_) and electrode parameters in the collected GITT profiles, the potassium-ion diffusion coefficient (D_K+_) is calculated. The calculated D_K+_ values during discharging (4.20 × 10^−13^~3.95 × 10^−12^ cm^2^ s^−1^) and charging (1.2 × 10^−12^~1.65 × 10^−11^ cm^2^ s^−1^) are significantly higher than micro-sized BiPO_4_ electrode (discharging: 7.8 × 10^−13^~1.82 × 10^−14^ cm^2^ s^−1^, charging: 1.2 × 10^−13^~3.5 × 10^−11^ cm^2^ s^−1^) (Figure 4f and Figure S12), also significantly exceeds some of the reported phosphate anodes [42,43,44]. The high K^+^ values indicate fast potassiation and depotassiation kinetics and excellent rate performance. The rapid potassium storage capacity of well-designed ultra-fine BiPO_4_@C electrodes can be attributed to their ingenious structural configuration, which provides an abundance of K^+^ insertion sites and a short K^+^ migration path, thereby accelerating the K^+^ reaction kinetics (as summarized in Figure S13).

### 2.4. Electrochemical Mechanism

**Figure 5:**
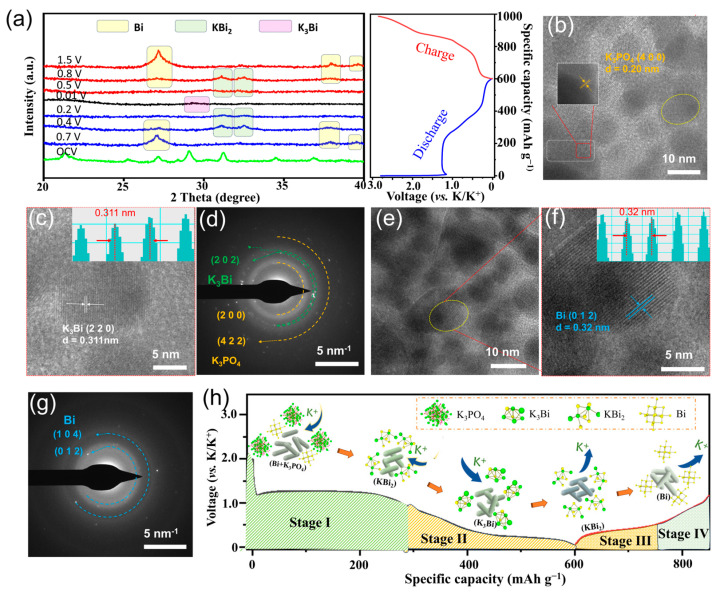
Investigation of the reaction mechanism of the BiPO_4_@C anode: (**a**) In-situ XRD and corresponding discharge-charge curve. (**b**,**c**) HRTEM images and (**d**) SAED pattern after discharge to 0.01 V. (**e**,**f**) HRTEM images and (**g**) SAED pattern after charge to 1.6 V. (**h**) Illustration of the reaction mechanism of the BiPO_4_@C anode.

Systematic ex-situ XRD and TEM testing were further employed to reveal the potassium storage mechanism of the BiPO_4_@C electrode. As depicted in Figure 5a, the BiPO_4_@C electrode exhibits a series of diffraction peaks located at 21.3°, 25.5°, 27.1°, 28.3°, 29.1°, 31.2°, 34.4°, 36.8°, and 36.8° at open circuit voltage (OCV), which are attributed to the (−111), (020), (200), (002), (120), (−112), (−202), and (112) crystal planes of monoclinic BiPO_4_. As the discharge progresses, the diffraction peak of BiPO_4_ gradually diminishes, and three characteristic peaks (located at 27.3°, 38.1°, and 39.8°) corresponding to the metal Bi emerges at 0.7 V, indicating a conversion reaction of BiPO_4_ (BiPO_4_ → Bi). Subsequently, the metal Bi is gradually alloyed into KBi_2_ (31.1° and 32.6°) at 0.4 V and further converted to K_3_Bi (28.6°) at 0.01 V (Bi → KBi_2_ → K_3_Bi). In the reverse charge process, the derivative peak of KBi_3_ gradually disappears and converts into KBi_2_ at 0.5 V before ultimately converting back to metallic Bi at 1.5 V, suggesting a gradual dealloying process (K_3_Bi → KBi_2_ → Bi). Subsequently, the ex-situ TEM test results provided further confirmed the experimental findings. Figure 5b,c clearly shows that the BiPO_4_@C electrode produced uniform nanoparticles when discharged to 0.01 V, with crystal plane spacings of 0.20 and 0.311 nm, corresponding to the (400) crystal plane of K_3_PO_4_ and the (220) crystal phase of K_3_Bi, respectively. Meanwhile, the (202) and (104) crystal planes of K_3_Bi and the (200) and (422) crystal planes of K_3_PO_4_ were identified by selected area electron diffraction (SAED) (Figure 5d). Upon reverse charging to 1.6 V, ultrafine metallic Bi particles belonging to the (012) and (014) crystal planes were successively detected (Figure 5e–g), thereby demonstrating the excellent reversibility of the stepwise alloying-dealloying reaction of the BiPO_4_@C electrode. To sum up, the in-situ/ex-situ test results combined with the CV curves reveal the reversible conversion and stepwise alloying/dealloying reaction mechanism of the BiPO_4_@C electrode. The detailed evolution process of the mechanism is illustrated in Figure 5h and described as follows:

Stage I: (OCV–0.8 V, Conversion reaction and the formation of SEI layer):

BiPO_4_ + 3 K^+^ + 3 e^−^ → K_3_PO_4_ + Bi

Stage II: (0.8–0.01 V, Step-alloying reaction):

2 Bi + K^+^ + e^–^ → KBi_2_

KBi_2_ + 5 K^+^ + 5 e^−^ → 2 K_3_Bi

Stage III: (0.01–0.5 V, Dealloying reaction):

2 K_3_Bi − 5 K^+^ − 5 e^−^→ KBi_2_

Stage IV: (0.5–1.6 V, Dealloying reaction):

KBi_2_ − K^+^ − e^–^→ 2 Bi

### 2.5. Application Potential

To fully demonstrate the application potential of BiPO_4_@C, we have re-prepared thick electrodes (about 4.0 mg/cm^2^) and investigated their potassium storage performance. As shown in Figure S14, the BiPO_4_@C thick electrode showed high specific capacity and superior cycling stability, maintaining 218.8 mAh g^–1^ after 80 cycles at 50 mA g^–1^ (Figure S14a), 180.9 mAh g^–1^ after 200 cycles at 200 mA g^–1^ (Figure S14b), and 130.3 mAh g^–1^ after 80 cycles at 1000 mA g^–1^ (Figure S14c). Impressively, the thick electrode demonstrated excellent rate performance, with capacities of 280.1, 219.9, 194.8, 152.4, and 102.6 mAh g^–1^ at 50–1000 mA g^–1^, respectively (Figure S14d). Compared to the commercial BiPO_4_ thick electrode, the BiPO_4_@C thick electrode has significantly better structural advantages, as evidenced by its specific capacity, capacity retention rate and rate performance at various current densities (Figure S15). Overall, the BiPO_4_@C electrodes maintained excellent cycling stability and rate performance even at the increased loading mass of 4.0 mg cm^–2^, which also confirms its potential for commercialization. The excellent performance is attributed to the nanosized BiPO_4_ significantly reducing the volume strain and increasing the embedded potassium sites; the incorporation of the bulk and open-skeleton anion PO_4_^3−^ serves to mitigate the considerable volume distortion and generates the high ionic conductivity product (K_3_PO_4_) to ameliorate the poor ionic transport due to the structural deformation.

## 3. Experimental

### 3.1. Sample Preparation

Synthesis of BiPO_4_@C nanorods. All chemicals were analytical reagent grade and used as received without further treatment. BiPO_4_@C nanorods were synthesized using a typical solvothermal method. First, 0.5 mmol Bi(NO_3_)_3_ (99%, Aladdin Reagent Co., Ltd., Shanghai, China) and 0.5 mmol NH_4_H_2_PO_4_ (99%, Aladdin Reagent Co., Ltd., China) were dissolved in 30 mL of ethylene glycol (99%, Aladdin Reagent Co., Ltd., China) with stirring for 6 h. Then, 1 mL of deionized (DI) water was added and stirred rapidly for 5 min. The mixture was transferred to a 50 mL autoclave and heated at 160 °C for 12 h. The BiPO_4_ nanorods were washed six times by centrifugation with ethanol and DI water. Finally, they were dried overnight at 80 °C.

### 3.2. Electrochemical Measurements

The working electrodes were prepared by mixing active materials, Super P, and polyvinylidene fluoride (PVDF) in a weight ratio of 7:2:1 with N-methyl-2-pyrrolidone (NMP) as solvent. The CR2032 half-cells were assembled with K-metal, glass fiber (Whatman), and 3 M potassium bis(fluorosulfonyl)imide (KFSI) in dimethyl ether (DME) as the counter electrodes, the separators, and the electrolyte, respectively. Cyclic voltammetry (CV) and electrochemical impedance spectroscopy (EIS, frequency range from 1.0 × 10^5^ to 0.1 Hz) tests were performed on an Autolab instrument (PGSTAT 302). Galvanostatic charge-discharge tests were performed on a Neware battery tester (Neware CT-4008). The mass loading of the active materials (BiPO_4_@C) was about 1.2 mg cm^−2^. And the specific capacity was based on the mass of BiPO_4_@C.

## 4. Conclusions

In summary, we have integrated the dual strategies of architecture engineering and anion tuning to construct an ultrathin carbon-coated BiPO_4_ nanorods (BiPO_4_@C) electrode to suppress volume distortion and unclog electron transport pathways. The ultrathin nanorods provided a substantial number of electrochemical reaction sites, negated the size-related volume strain effect, and also shortened the ion transport distance. Meanwhile, the incorporation of an ultrathin carbon layer and bulk PO_4_^3−^ served to defend and buffer the volume expansion associated with the alloying-dealloying process, thereby markedly enhancing the cycling stability of the BiPO_4_@C anode. Significantly, the in-situ generated K_3_PO_4_ with high ionic conductivity acts as a bridge to unblock the electron/ion transport pathway and significantly improves the rate performance. Moreover, the preparation and testing of thick electrodes confirmed the excellent electrochemical performance and application potential of BiPO_4_@C electrodes. Thus, this work highlights the significance of architecture engineering and anion-tuning strategy in rationally designing high-performance electrodes for K-ion batteries.

## Data Availability

The dataset is available upon request from the authors.

## Supplementary Materials

The following supporting information can be downloaded at: https://www.mdpi.com/article/10.3390/molecules30030729/s1, Figure S1: SEM images of BiPO_4_@C composites with different raw material concentrations; Figure S2: SEM images of BiPO_4_@C composites at different reaction temperatures; Figure S3: SEM images of BiPO_4_@C composites at different reaction times; Figure S4: XRD patterns and Rietveld refinement plots of BiPO_4_@C; Figure S5: XRD pattern of BiPO_4_@C residue after the TGA test; Figure S6: SEM image and XRD patterns of commercial micro-sized BiPO_4_; Figure S7: Comparison of rate performance between BiPO_4_@C anode and the previously reported Bi-based compound anode in PIBs.; Figure S8: Cycling performances of (a) BiPO_4_@C and (b) commercial micro-sized BiPO_4_ anodes at a current density of 1.0 A g^−1^; Figure S9: Surface and cross-sectional SEM images of the BiPO_4_@C electrode after 100 cycles; Figure S10: Surface and cross-sectional SEM images of the BiPO_4_ electrode after 100 cycles; Figure S11: EIS results of the (a) BiPO_4_@C electrode and (b) commercial micro-sized BiPO_4_ electrode before and after different cycles at 500 mA g^−1^; Figure S12: (a) GITT curves, (b) the corresponding detailed voltage response in a single current pulse, and the K^+^ diffusion coefficient values (D_K+_) during the (c) discharge and (d) charge process; Figure S13: Schematic illustration of potassium-ion diffusion in the nanosize structure; Table S1: Crystallographic parameters from Rietveld refinement for the XRD pattern of BiPO_4_@C.
