# Supplementary material for: Synergistic Effect of Anionic-Tuning and Architecture Engineering in BiPO4@C Anode for Durable and Fast Potassium Storage
Source: Molecules. 2025 Feb 6;30(3):729. doi: 10.3390/molecules30030729 (PMC11820522; doi:10.3390/molecules30030729)
Supplement: Supplementary file 1 [file molecules-30-00729-s001.zip › molecules-3367638-supplementary.pdf]

# Synergistic effect of anionic-tuning and architecture engineering in $BiPO_4$@C anode for durable and fast potassium storage

*Heying Chu [1], Yong Li [1], Yuanjie Liu [1], Xueping Chai [1], Hongzhou Zhang [1,]* and Jingchuan Zhang [1,]**

[1] College of Mechanical and Electronic Engineering, Tarim University, Xinjiang, Alar, 843300, China

* Corresponding authors:

shenzhouxing@taru.edu.cn (H.Z.Z); zhangjingchuan@taru.edu.cn (J.C.Z.)

## Section SI. Experimental Section

**Material Characterization.** The crystal structure and morphology of the samples was characterized by X-ray diffraction (XRD, SmartLab9KW, 40 kV, Cu Kα radiation, $\lambda$ = 0.154 nm), scanning electron microscopy (SEM, Thermo Scientific Apreo C, USA) and transmission electron microscopy (TEM, Talos F200S, FEI, Thermo). The $BiPO_4$ and carbon contents of the sample were analyzed by a thermal analyzer (TGA, DSC 3+, Mettler Toledo). X-ray photoelectron spectroscopy (XPS) analysis and Fourier transform infrared spectroscopy (FTIR, Bruker MPA and Tensor 27) was also used. Raman spectroscopy was performed on a Raman spectrometer (Renishaw InVia, 785 nm excitation wavelength).

### Calculation process for the capacitance effect and pseudocapacitive contribution

The capacitance effect can be determined from the CV curves at different scan rates, according to the relationship between measured peak currents ($i$) and scanning rates ($v$), as follows:

$$i = a\, v^{b}, \qquad \text{(S1)}$$

$$\log i = b \log v + \log a, \qquad \text{(S2)}$$

where $a$ and $b$ are the fitting parameters, and $i$ and $v$ represent peak current and scanning rate, respectively. The variable parameter $b$ is deduced from the slope of log ($i$)/log ($v$) within the range of 0.5–1.0. The electrochemical reaction is controlled by ion diffusion if $b$ approaches 0.5, while a value near 1.0 means the dominance of capacitive behavior

in the electrochemical reaction [1].

Furthermore, the capacitive contribution at various scan rates can be quantitatively calculated via the following equation with the parameters $k_1$ and $k_2$:

$$I = k_1\, v + k_2\, v^{1/2}, \qquad \text{(S3)}$$

where $k_1\, v$ and $k_2\, v^{1/2}$ represent the contributions of the capacitive and diffusion behaviors in the reactions, respectively [2].

**Calculation process for the diffusion coefficient ($D_{Na+}$)**

The galvanostatic intermittent titration technique (GITT) tests were performed by discharging or charging the cells for 30 min at 0.02 A $g^{-1}$, followed by a 1.0 h relaxation in the voltage range of 0.01 to 1.5 V. The $D_{Na+}$ can be worked out by solving Fick's second law according to the following equation.

$$D = \frac{4}{\pi\tau}\left(\frac{m_B V_M}{M_B S}\right)^2\left(\frac{\Delta E_S}{\Delta E_\tau}\right)^2\left(\tau \ll \frac{L^2}{D}\right), \qquad \text{(S4)}$$

where $\tau$ is the relaxation time (s), and $m_B$, $V_M$, $M_B$, and $S$ are the mass, the molar volume of the active material, the molar mass and the area of the electrode, respectively. $\Delta E_S$ represents the quasi-thermodynamic equilibrium potential difference between before and after the current pulse. $\Delta E_\tau$ is the potential difference during the current pulse [3].

## Section SII. Supplementary Figures and Tables

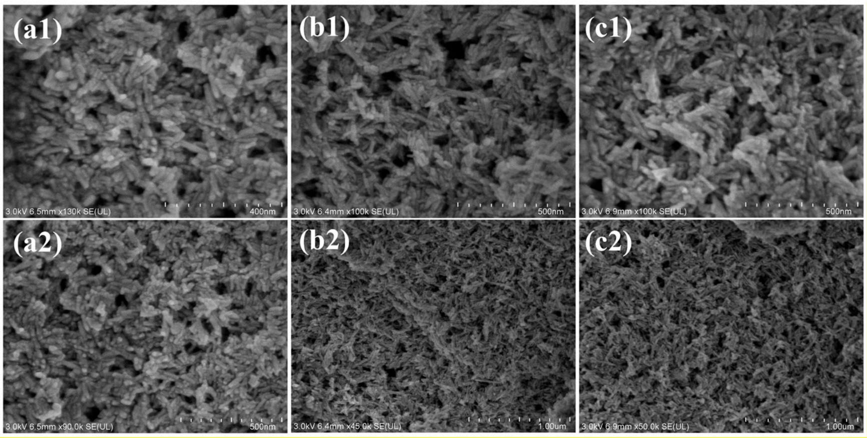


**Figure S1.** SEM images of $BiPO_4$@C composites with different raw material concentrations of (a) 0.2, (b) 0.5, and (c) 1.0 mmol.

Figure S1 shows the SEM images of $BiPO_4$@C nanorods with different multiples at reaction concentrations of 0.2, 0.5, and 1.0 mmol, respectively. The comparison results show that the diameter and length of the $BiPO_4$@C nanorods increased sequentially with rising concentration.

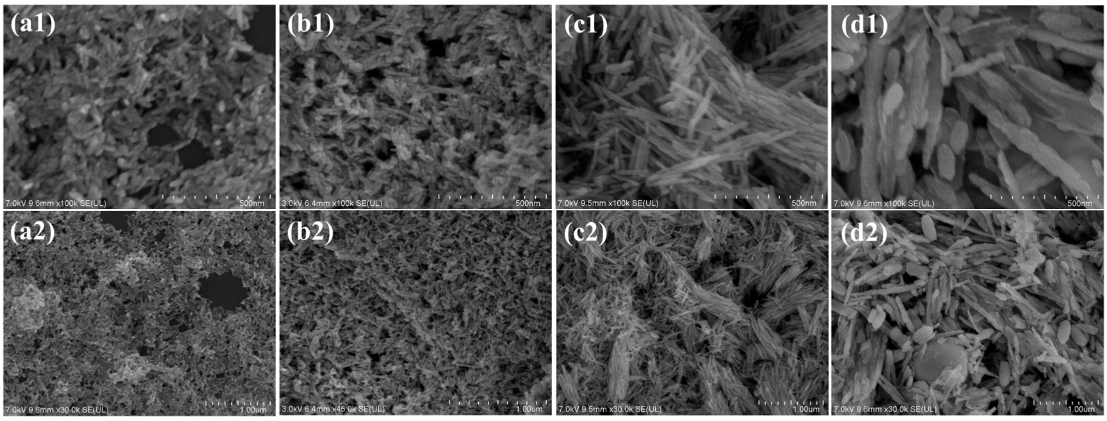


**Figure S2.** SEM images of $BiPO_4$@C composites at different reaction temperatures of (a) 140 °C, (b) 160 °C, (c) 180 °C, and (d) 200 °C.

Figure S2 depicts the SEM images of $BiPO_4$@C nanorods at different reaction temperatures of 140, 160, 180 and 200 °C, respectively. It is evident that the length of the $BiPO_4$@C nanorods increases in a sequential manner with the reaction temperature. The nanorods were transformed into rod-like clusters at 180 °C, and exhibited an irregular sheet-like structure at 200 °C.

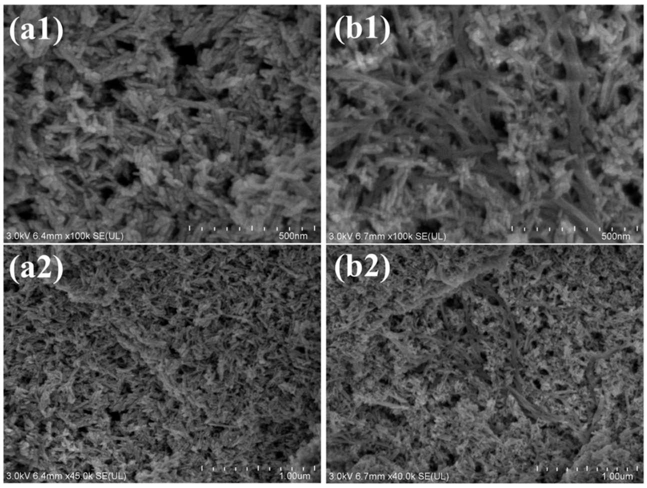


**Figure S3.** SEM images of $BiPO_4$@C composites at different reaction times of (a) 12 h and (b) 24 h.

With regard to the impact of reaction time, as illustrated in Figure S3, when the reaction time is extended to 24 h, the rod-like structure exhibits uncontrolled growth and ultimately assumes a dendritic configuration.

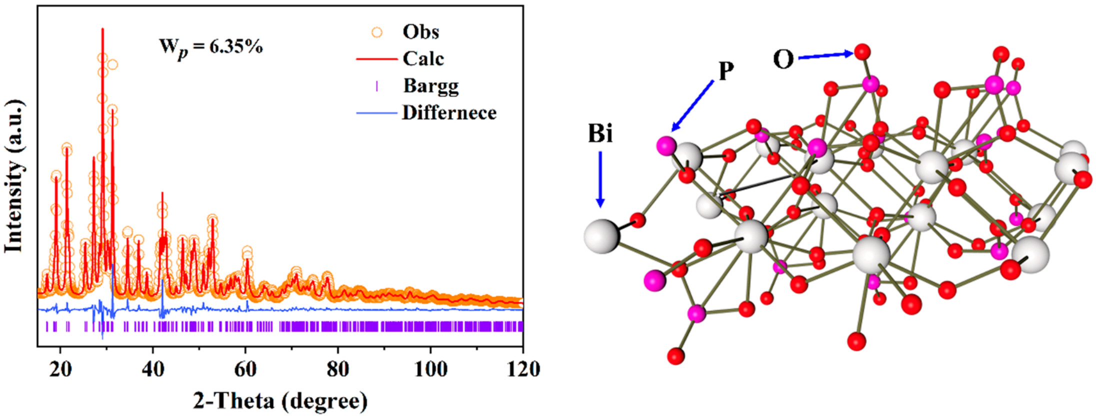


**Figure S4**. XRD patterns and Rietveld refinement plots of $BiPO_4$@C.

$BiPO_4$@C exhibits a monoclinic structure characterized by the space group $P2_1/n$ (PDF#04-010-5606). The lattice parameters of $BiPO_4$, obtained by Rietveld refinement, are as follows: a = 6.6658 Å, b = 6.8869 Å, c = 6.4148 Å, β = 103.79°. More details on the Rietveld refinements and structural parameters of $BiPO_4$@C are given in Table S1.

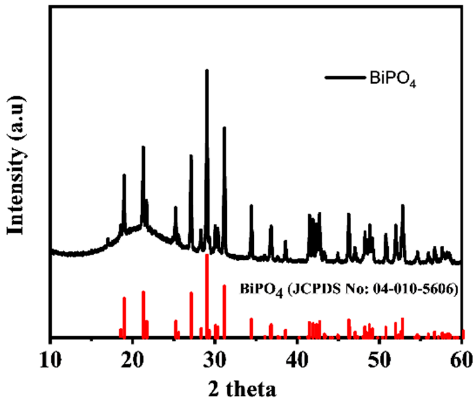


**Figure S5**. XRD pattern of BiPO4@C residue after the TGA test.

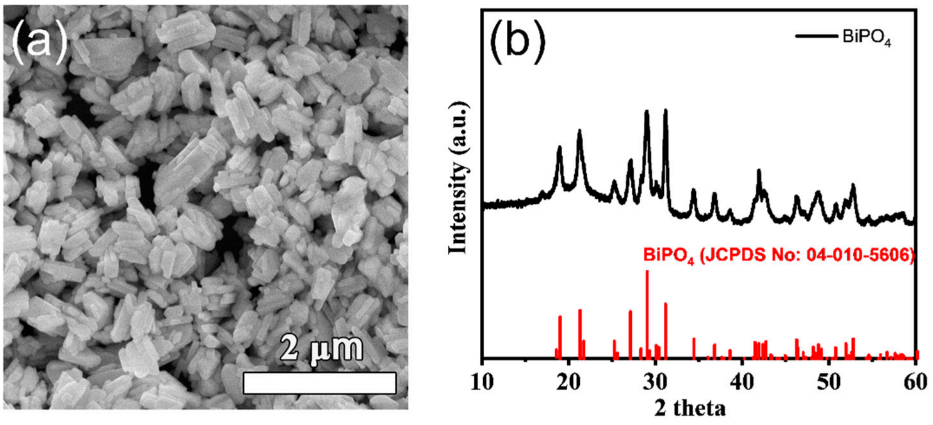


**Figure S6**. SEM image and XRD patterns of commercial micro-sized BiPO4.

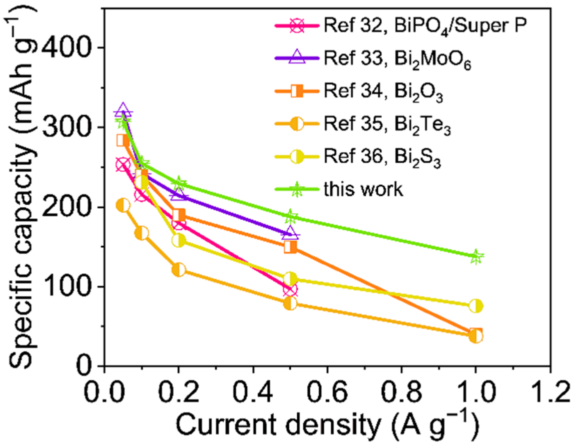


**Figure S7**. Comparison of rate performance between BiPO4@C anode and the previously reported Bi-based compound anode in PIBs.

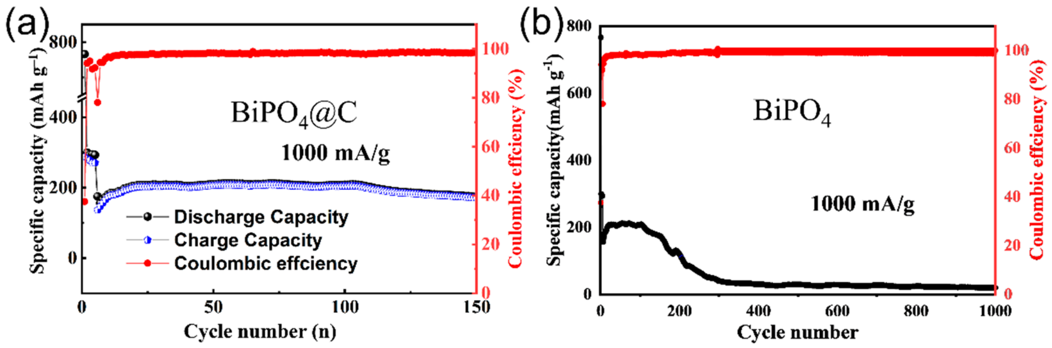


**Figure S8.** Cycling performances of (a) $BiPO_4$@C and (b) commercial micro-sized $BiPO_4$ anodes at a current density of 1.0 A $g^{-1}$.

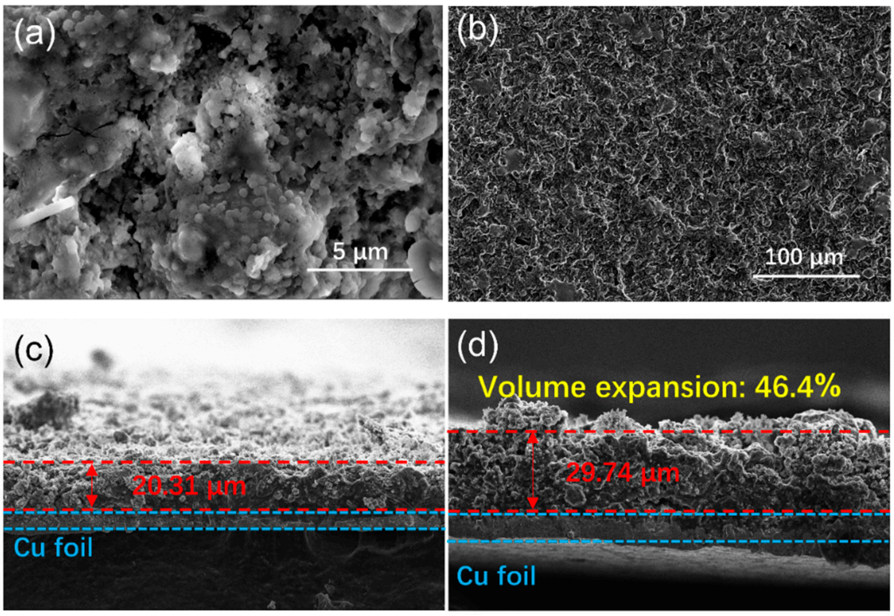


**Figure S9.** (a, b) Surface SEM images of the $BiPO_4$@C electrode after 100 cycles. Cross-sectional SEM images of the $BiPO_4$@C electrode (c) before and (d) after 100 cycles.

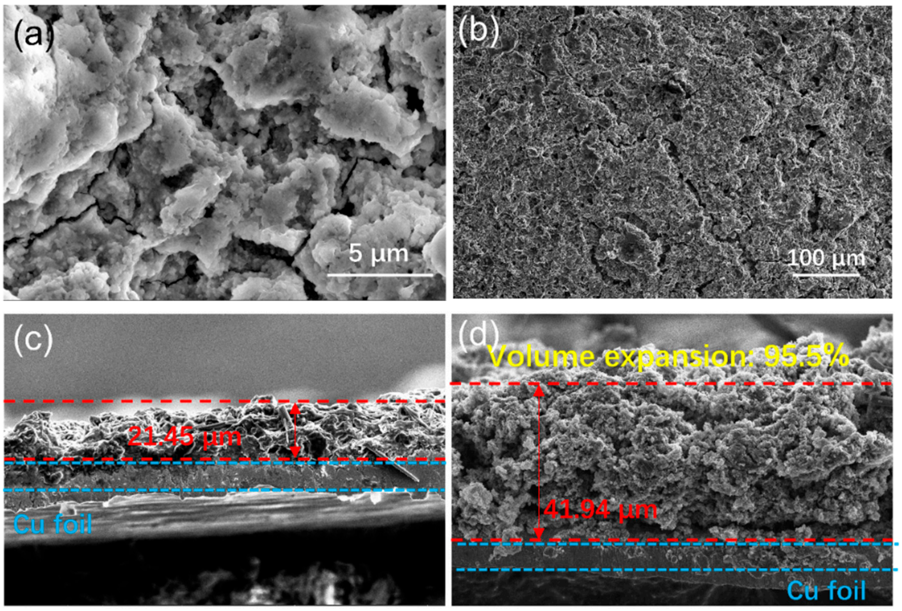


**Figure S10.** (a, b) Surface SEM images of the commercial micro-sized $BiPO_4$ electrode after 100 cycles. Cross-sectional SEM images of the commercial micro-sized electrode (c) before and (d) after 100 cycles.

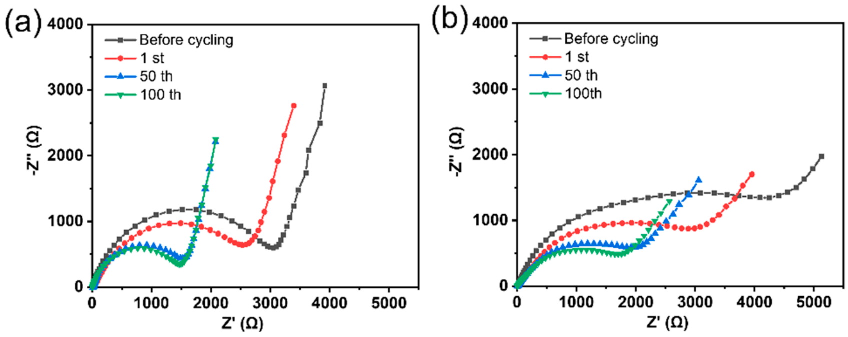


**Figure S11.** EIS results of the (a) $BiPO_4$@C electrode and (b) commercial micro-sized $BiPO_4$ electrode before and after different cycles at 500 mA $g^{-1}$.

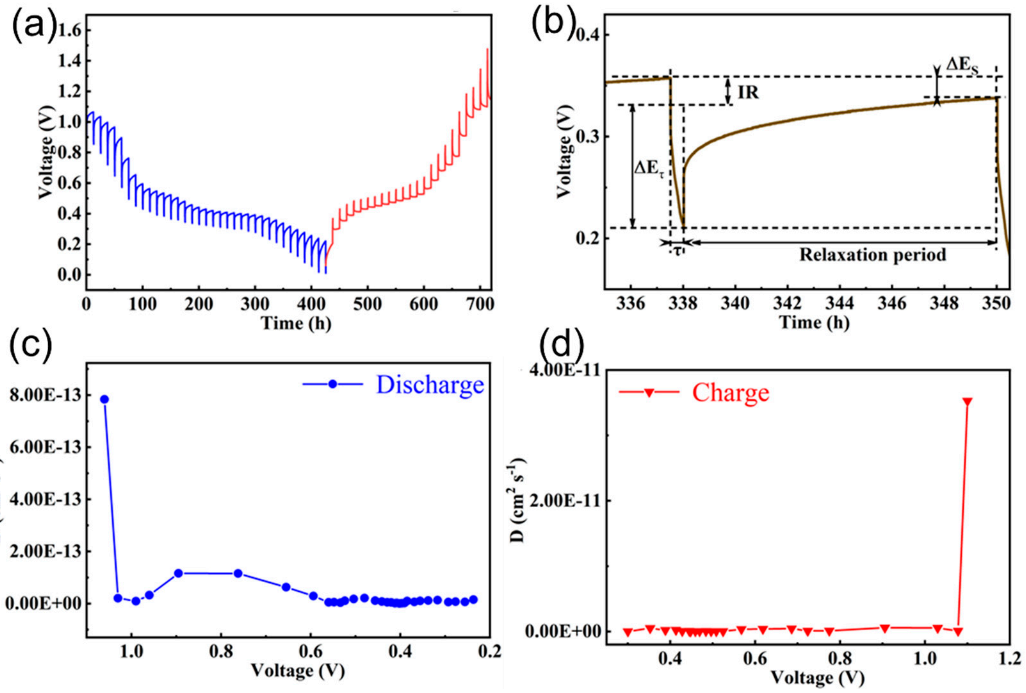


**Figure S12.** Kinetic analysis of the micro-sized $BiPO_4$ electrode. (a) GITT curves, (b) the corresponding detailed voltage response in a single current pulse, and the $K^+$ diffusion coefficient values ($D_{K+}$) during the (c) discharge and (d) charge process.

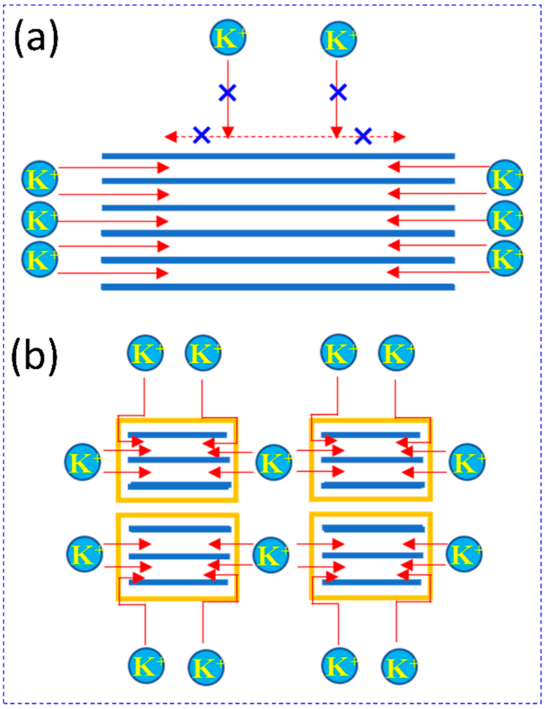


**Figure S13.** Schematic illustration of potassium-ion diffusion in the nano-size structure.

Specifically, the yellow square represents active substances with different sizes, and the blue lines represent lattice fringes. Large particles with long-range ordered lattice fringes mean longer paths for potassium-ion transport. Refined particles have abundant potassium-ion embedding sites and short-range potassium ion diffusion paths.

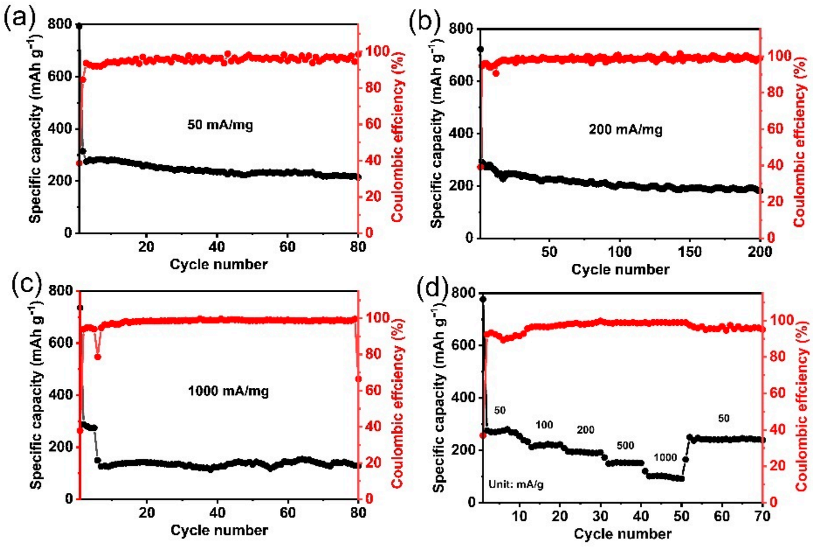


**Figure 14.** Cycling performances at (a) 50 mA/g, (b) 200 mA/g, (c) 500 mA/g, and (d) Rate performances of $BiPO_4$@C thick electrode.

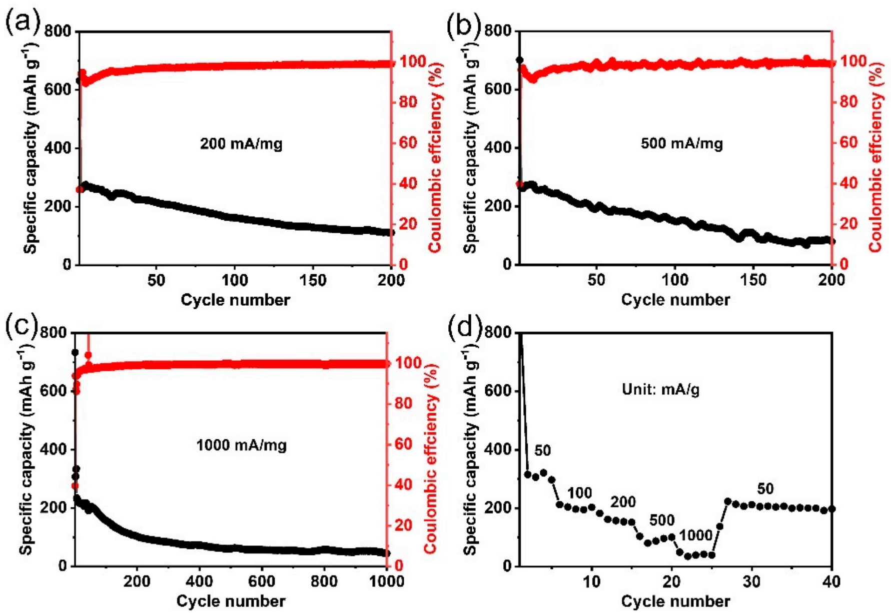


**Figure 15.** Cycling performances at (a) 200 mA/g, (b) 5000 mA/g, (c) 1000 mA/g, and (d) Rate performances of commercial $BiPO_4$ thick electrode.

**Table S1**. Crystallographic parameters from Rietveld refinement for the XRD pattern of $BiPO_4$@C.

| **Space group = P2₁/n, a = 6.6658(6) Å, b = 6.8869(15) Å, c = 6.4148(5) Å, β = 103.79°(21), V=294.997(8)** <br> **Rp=5.35%, Rwp=6.35%, RF=3.61%, χ2=3.34** | | | | | | |
|---|---|---|---|---|---|---|
| **Atom** | site | x | y | z | occupancy | Uiso |
| Bi | 4e | 0.2821(4) | 0.1482(4) | 0.0906(4) | 1 | 0.0127(8) |
| P | 4e | 0.2995(19) | 0.1631(20) | 0.6131(20) | 1 | 0.012(3) |
| O1 | 4e | 0.2614(4) | 0.0039(31) | 0.4422(4) | 1 | 0.023(9) |
| O2 | 4e | 0.3791(28) | 0.3412(4) | 0.5176(32) | 0.9 | 0.037(7) |
| O3 | 4e | 0.4674(29) | 0.1038(4) | 0.8169(26) | 1 | 0.045(7) |
| O4 | 4e | 0.1214(4) | 0.2059(5) | 0.7127(4) | 1 | 0.006(9) |

## REFERENCES


1. Li Q.H.; Yu, D.D.; Peng, J.; Zhang, W.; Huang, J.L.; Liang, Z.X.; Wang, G.Y.; Li, H.X.; Xiong, S.Y.; Wang, J.Z.; Huang, S.M. Efficient Polytelluride Anchoring for Ultralong-Life Potassium Storage: Combined Physical Barrier and Chemisorption in Nanogrid-in-Nanofiber, *Nano-Micro Letters.* **2024**, *16*, 77.

2. Deng, S.; Li, C.; Feng, W.; Cao, Y.; Tian, X.; Bi, H.; Zhou, S.; Wong, C.-P.; Dong, Y. Ultra-small ZnS enhanced by Fe-N-C for advanced potassium-ion hybrid capacitors: Electronic transfer dynamics and ion adsorption capability. *Nano Energy* **2023**, *106*, 108065.

3. Sha, M.; Liu, L.; Zhao, H.; Lei, Y. Anode materials for potassium-ion batteries: Current status and prospects. *Carbon Energy*. **2020**, *2*, 350−369.
